# Efficacy of disitamab vedotin in non-small cell lung cancer with *HER2* alterations: a multicenter, retrospective real-world study

**DOI:** 10.3389/fonc.2024.1441025

**Published:** 2024-11-06

**Authors:** Meiling Zhang, Li Wang, Qian Wang, Jiu Yang, Wei Peng, Xiaoyou Li, Meiqi Shi, Kaihua Lu

**Affiliations:** ^1^ Department of Oncology, The First Affiliated Hospital of Nanjing Medical University, Nanjing, China; ^2^ Department of Medical Oncology, Jiangsu Cancer Hospital & Jiangsu Institute of Cancer Research & The Affiliated Cancer Hospital of Nanjing Medical University, Nanjing, China

**Keywords:** *HER2* mutation, *HER2* amplification, target, non-small cell lung cancer, RC48

## Abstract

**Background:**

Non-small cell lung cancer (NSCLC) with human epidermal growth factor receptor 2 (*HER2*) alterations poses a substantial treatment challenge. Current *HER2*-targeted therapies offer limited efficacy. Antibody-drug conjugates (ADCs) targeting *HER2* have emerged as a promising therapeutic strategy. This study aimed to evaluate the clinical response to a novel ADC drug Disitamab vedotin (RC48) in advanced NSCLC with *HER2* alterations.

**Methods:**

This study conducted a retrospective review of patients harboring *HER2* alterations treated with RC48 in the real world. Clinical outcomes were evaluated in terms of objective response rate (ORR), disease control rate (DCR), and progression-free survival (PFS).

**Results:**

Out of 22 patients, 21 (95.5%) received RC48 combination therapy, while one received RC48 monotherapy. The ORR of all patients reached 45.5%, and the DCR stood at 90.9%. The median PFS (mPFS) was 7.5 months. Among patients receiving RC48 combination therapy, the ORR was 47.7%, and the mPFS of 8.1 months. The combination of RC48 with platinum+/- bevacizumab resulted in the highest ORR of 71.4% (5 out of 7 patients), with *HER2* TKI following at a 50.0% ORR (4 out of 8 patients). First-line (1L) treatment with RC48 showed an ORR of 62.5% (5 out of 8 patients), second-line (2L) treatments had a 57.1% ORR (4 out of 7 patients), and beyond second-line (>2L) treatments exhibited a 14.3% ORR (1 out of 7 patients). Patients with 1L, 2L, or >2L treatment had a mPFS of 8.1 months, 7.2 months, and 7.4 months, respectively. Patients with *HER2* mutations or amplifications, and those with concurrent mutations and amplifications at baseline, showed mPFS of 8.1 months, 9.4 months, and 7.4 months, respectively. The mPFS was significantly longer in patients with *HER2* amplification. The most common adverse events included hand-foot syndrome (54.5%), asthenia (50.0%), decreased white blood cell count (45.5%), and liver impairment (45.5%). Grade 3 adverse events occurred in one (4.5%) patient.

**Conclusion:**

RC48, particularly in combination regimens, demonstrates promising efficacy in advanced NSCLC with *HER2* alterations. These findings underscore the need for further research to validate RC48’s application in clinical practice.

## 1 Introduction

Non-small cell lung cancer (NSCLC) with human epidermal growth factor receptor 2 (*HER2*) alterations mainly manifest as protein overexpression, gene amplification, or gene mutation (1–3). *HER2* mutations are found in 1-4% of NSCLC and amplifications are found in 2–5% of cases (4, 5). In comparison to other oncogenic drivers, *HER2* is a distinctive molecular with a poor prognosis (3, 6). The standard first-line treatment for advanced NSCLC with *HER2* alterations, immune checkpoint inhibitor (ICI) therapy, has shown limited clinical activity with an objective response rate (ORR) ranging from 7.4% to 27.3% and median progression-free survival (mPFS) ranging from 1.9 to 2.5 months (7). Tyrosine kinase inhibitors (TKIs) are transformative agents for the treatment of NSCLC, especially in terms of epidermal growth factor receptor (*EGFR*) and anaplastic lymphoma kinase (*ALK*). However, *HER2*-targeted TKIs such as afatinib (8, 9), poziotinib (10, 11), and pyrotinib (12) had moderate efficacy as second- or later-line therapies, with ORRs of 19–30% and mPFS 4.0-6.9 months.

Regarding *HER2*-targeted monoclonal antibodies, previous studies have mostly focused on NSCLC with *HER2* protein over-expression but they have shown limited efficacy (13–15). Antibody-drug conjugates (ADCs), consisting of a monoclonal antibody (mAb) carrying a high-activity cytotoxic drug (payload) via a chemical linker, are one of the fastest growing oncology therapeutics, and are now one of the potential options for lung cancer patients (16, 17). Currently, *HER2* ADCs such as trastuzumab deruxtecan (T-DXd) and ado-trastuzumab emtansine (T-DM1) have shown considerable clinical benefits. Both agents have been recommended as options for *HER2*-mutant NSCLC after progressing with standard treatment by the National Comprehensive Cancer Network (NCCN) guidelines (18). A phase II basket trial of T-DM1 showed an ORR of 44% and mPFS of 5 months in 18 patients with advanced *HER2*-mutant NSCLC patients (19). Another clinical trial reported a 51% ORR for T-DM1 in 49 patients with *HER2*-amplified or -mutant lung cancers (20). However, the efficacy of T-DM1 has not been validated in large-scale samples and has not been approved by the Food and Drug Administration (FDA). The pivotal DESTINY-Lung 02 trial of T-DXd reported a 49% ORR, 9.9 months mPFS, and 19.5 months median overall survival (mOS) in *HER2*-mutant NSCLC (21). Based on this data, the FDA approved 5.4mg/kg T-DXd for the treatment of *HER2*-mutant locally advanced or metastasis NSCLC in August 2022. Nevertheless, 13% of patients treated with T-DXd developed adjudicated drug-related interstitial lung disease (2.0% grade >3), and one patient developed ILD at grade 5, which limits its widespread use in NSCLC patients.

Disitamab vedotin (RC48) emerges as an innovative therapeutic agent, consisting of a humanized anti-*HER2* antibody linked to monomethyl auristatin E (MMAE) via a cleavable linker (22). The National Medical Products Administration of China (NMPA) has approved RC48 for patients with *HER2*-overexpressing metastatic gastric cancer/gastroesophageal junction (G/GEJ) adenocarcinoma after >2L of treatment, and *HER2* IHC2+/3+ metastatic urothelial carcinoma post-platinum-based therapy. To date, RC48 has demonstrated promising antitumor activity and a manageable safety profile in clinical applications.

The purpose of this study is to explore the efficacy and safety of RC48 with unresectable locally advanced or metastatic NSCLC patients harboring *HER2* mutations or amplifications.

## 2 Materials and methods

### 2.1 Study design and patient population

We conducted a retrospective observational study at The First Affiliated Hospital of Nanjing Medical University (Jiangsu Provincial People’s Hospital) and Jiangsu Cancer Hospital & Jiangsu Institute of Cancer Research & The Affiliated Cancer Hospital of Nanjing Medical University, from August 2021 to March 2023. Patients over 18, diagnosed pathologically with NSCLC of unresectable, locally advanced, or metastatic stage, and confirmed to have *HER2* mutations or amplifications via PCR or NGS, were included. Data cutoff date of July 30th, 2023. Our investigation included a comprehensive review of clinicopathological characteristics, encompassing demographic data, smoking status, ECOG-PS score, cancer stage, and histological type, along with *HER2* genomic alteration status. The specifics of the treatment combination therapies, such as the dosage, treatment cycles, and duration, were documented. Ethical approvals were obtained in Ethical Committees from both institutions.

Of the 40 patients initially screened, patients with incomplete medical records, lacking follow-up, or without documented *HER2* genomic status were excluded. Eventually 22 eligible cases were enrolled in this study.

### 2.2 Efficacy assessment of treatment

Anonymized data were evaluated for clinicopathologic characteristics and outcomes for RC48 treatment, focusing on ORR, disease control rate (DCR), and PFS. Objective responses were evaluated based on Response Evaluation Criteria in Solid Tumors (RECIST, v1.1), where ORR was defined as the percentage of patients achieving either a complete response (CR) or partial response (PR) to the treatment. DCR was calculated as the proportion of patients exhibiting a CR, PR, or stable disease (SD). PFS was defined as the duration from the onset of treatment to the occurrence of disease progression or death from any cause. Patients experiencing relapse within six months post-systemic anticancer therapy were subsequently classified as receiving second-line treatment for their advanced disease.

### 2.3 Statistical analysis

For continuous variables, medians and ranges were used to summarize the demographic and clinical characteristics of patients, whereas for categorical variables, frequencies and percentages were used to describe them. The Kaplan-Meier method was employed to analyze survival outcomes. To investigate the impact of different treatments on PFS among various patient subgroups, univariate analyses were conducted. The log-rank test was employed to assess the significance of differences in PFS, with a threshold of P < 0.05 for statistical significance. All analyses were performed using R software (version 3.5.1).

## 3 Results

### 3.1 Patient characteristics

A total of 22 patients with *HER2*-altered NSCLC receiving monotherapy or combination therapy with RC48 were enrolled from August 2021 and March 2023. A significant majority, representing 90.9% (20 out of 22), had adenocarcinoma histologically. Only a single patient (4.5%) was treated with RC48 as a monotherapy, whereas the remaining 21 (95.5%) received combination therapies. Detailed therapeutic regimens included 8 patients with TKIs, 7 with platinum with or without bevacizumab (3 only with platinum, 4 with platinum combined with bevacizumab), 4 with antiangiogenic drugs, 2 with PD-(L)1 inhibitor with or without bevacizumab. Further demographic and clinical characteristics of the patients are shown in **Table 1**. Notably, eight (36.4%) patients received RC48 as 1L treatment, while 2L or >2L treatments were received by 7 (31.8%) each. Molecular profiling performed at baseline disclosed 15 patients with *HER2* mutation, 5 with *HER2* amplifications, and 2 harboring both mutation and amplification simultaneously. Brain metastases were observed in 31.8% of the patients.

**Table 1:** Patients baseline characteristics and treatment therapies.

Characteristics	N=22
Sex, n (%)	
Male	14 (63.6)
Female	8 (36.4)
Age, median (range), years	61 (45-82)
Smoking history, n (%)	
Never	8 (36.4)
Former	14 (63.6)
Histology, n (%)	
Adenocarcinoma	20 (90.9)
NSCLC-nos	2 (9.1)
Brain metastases, n (%)	
No	15 (68.2)
Yes	7 (31.8)
Stage, n (%)	
III	4 (18.2)
IV	18 (81.8)
ECOG PS score, n (%)	
0	9 (40.9)
1	13 (59.1)
*HER2* alteration status, n (%)	
Mutation	15 (68.2)
Amplification	5 (22.7)
Concurrent amplification and mutation	2 (9.1)
*HER2* mutation, n (%)	
Y772_A775dup (exon20)	6 (50.0)
G776delinsVC (exon20)	1 (8.3)
771insAYVM (exon20)	1 (8.3)
A775_G 776insYVMA (exon20)	1 (8.3)
V777_G778insGSP (exon20)	3 (25.0)
V659E (exon17)	1 (33.3)
p.S310Y (exon8)	1 (33.3)
p.L755S (exon19)	1 (33.3)
unknow (exon20)	2 (11.8)
*HER2* Amplification, median (range), copy number gain	6.8 (3.0-9.0)
RC48 treatment line, n (%)	
1L	8 (36.4)
2L	7 (31.8)
>2L	7 (31.8)
Prior anti-PD- (L)1 therapy, n (%)	7 (31.8)
Prior anti-HER2 therapy, n (%)	7 (31.8)
RC48 treatment regimen, n (%)	
RC48 alone	1 (4.6)
RC48 combination therapy	21 (95.5)
RC48+ *HER2* TKIs	8 (38.1)
RC48+ Afatinib	1 (4.8)
RC48+ Pyrotinib	7 (33.3)
RC48+ platinum +/- bevacizumab	7 (33.3)
RC48+Carboplatin	2 (9.5)
RC48+ Loroplatin	1 (4.8)
RC48+Carboplatin + bevacizumab	3 (14.3)
RC48+Loroplatin+ bevacizumab	1 (4.8)
RC48+ antiangiogenic drugs	4 (19.1)
RC48+bevacizumab	1 (4.8)
RC48+Anrotinib	3 (14.3)
RC48+ PD- (L)1 inhibitors +/- bevacizumab	2 (9.5)
RC48+PD- (L)1 inhibitors	1 (4.8)
RC48+PD- (L)1 inhibitors + bevacizumab	1 (4.8)
RC48 dosing cycle, n (%)	
2 weeks	1 (4.6)
3 weeks	10 (45.5)
4 weeks	11 (50.0)

*HER2*, human epidermal growth factor receptor 2; NSCLC, non-small cell lung cancer; ECOG, Eastern Cooperative Oncology Group; PS, performance status; TKI, tyrosine-kinase inhibitor; PD-1, programmed cell death-1; PD-L1, programmed cell death-ligand1; 1L, first line; 2L, second line.

### 3.2 Efficacy

**Figure 1:**
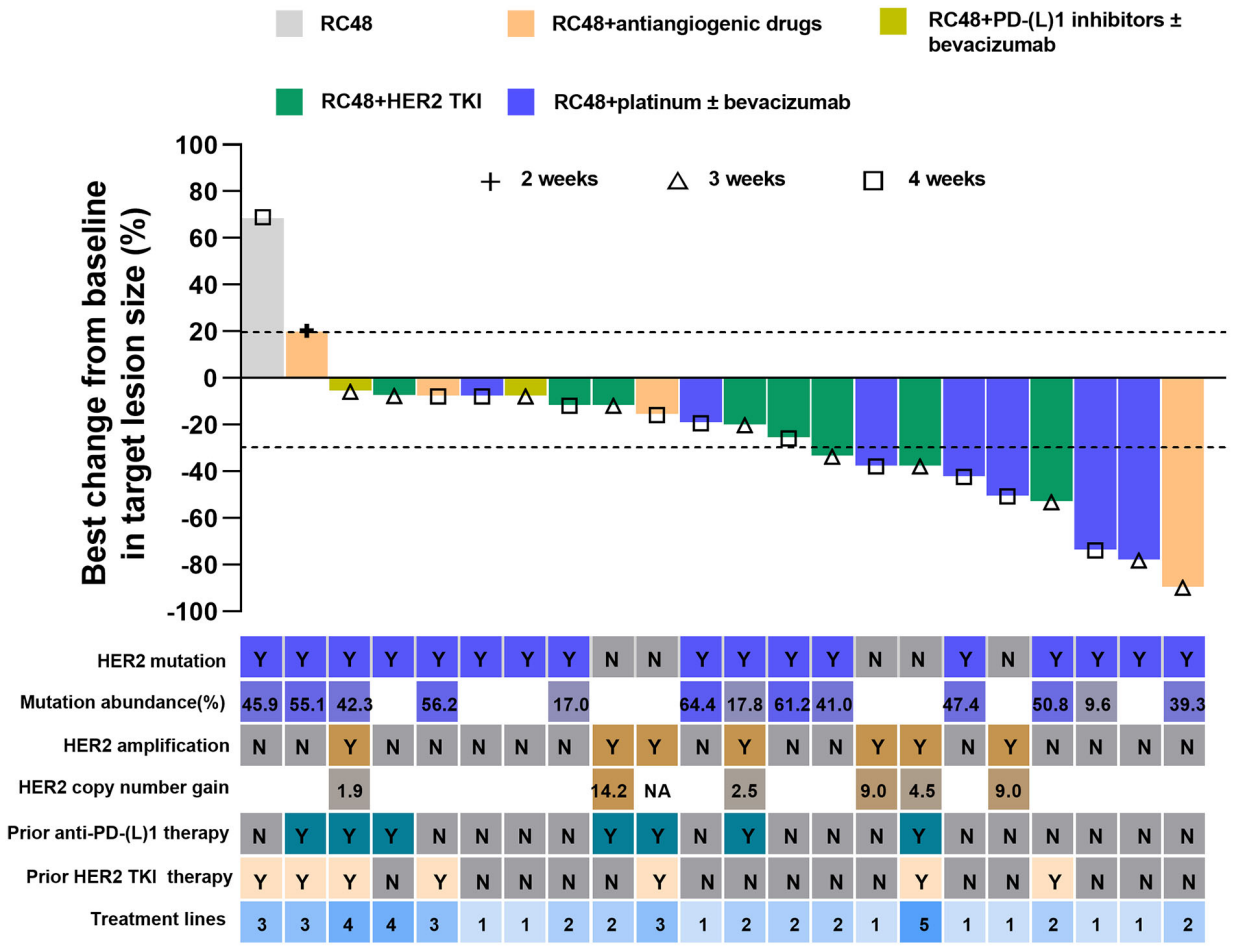
Best change from baseline in target lesion size by each patient. The line at -30% indicates a partial response. Alphabet in the *HER2* mutation row or *HER2* amplification row indicate status. Y, yes; N, no; *HER2*, human epidermal growth factor receptor 2; TKI, tyrosine kinase inhibitor; PD-1, programmed cell death-1; PD-L1, programmed cell death-ligand1; CI, Confidence interval.

**Figure 2:**
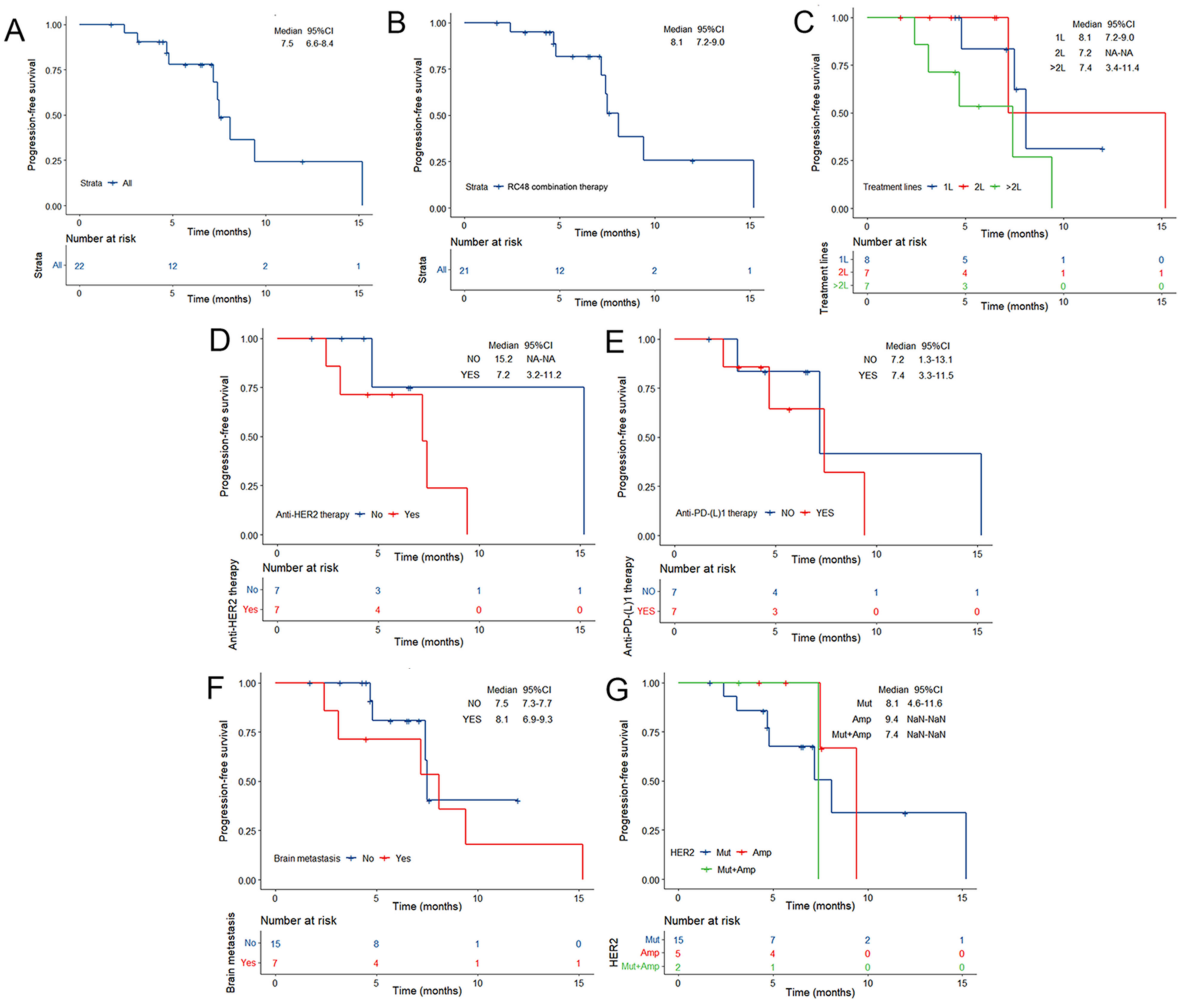
Kaplan–Meier estimates of PFS according to **(A)** the overall NSCLC population, **(B)** RC48 combination therapy, **(C)** RC48 treatment line, **(D)** prior anti-*HER2* therapy, **(E)** prior anti-PD-(L)1 therapy, **(F)** brain metastases and **(G)** *HER2* alteration status at baseline. TKI, tyrosine kinase inhibitor; PD-1, programmed cell death-1; PD-L1, programmed cell death-ligand1; CI, Confidence interval. Mut, mutation; Amp, amplification; Mut+ Amp, concurrent mutation and amplification; HER, human epidermal growth factor receptor 2; CI, Confidence interval.

Of the 22 patients, 10 (45.5%) patients achieved PR, and 10 (45.5%) patients showed SD, with a confirmed investigator-assessed ORR of 45.5% (10 out of 22) and a DCR of 90.9% (20 out of 22). A waterfall plot for the best percentage change in target lesion size is shown in **Figure 1**. At the time of data cut-off, survival analysis was conducted on all 22 patients, with the mPFS of 7.5 months (95% CI, 6.6-8.4 months) and the estimated 6-month PFS rate and 12-month PFS rate of 77.9% and 24.4%, respectively (**Figure 2A**).

**Figure 3:**
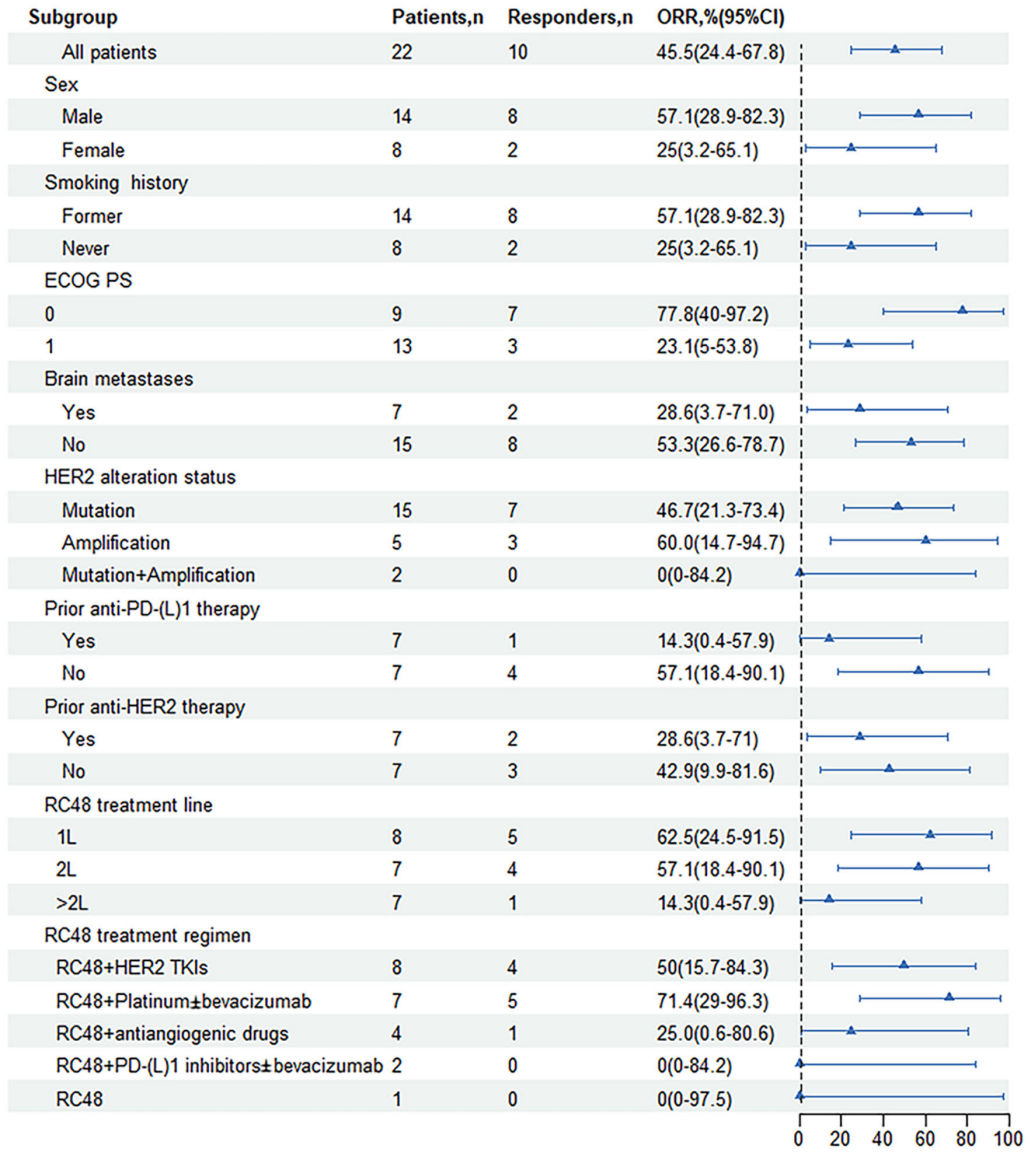
Forest plot of subgroup analysis of objective response rates by baseline demographic and disease characteristics. ORR, objective response rate; ECOG, Eastern Cooperative Oncology Group; PS, performance status; *HER2*, human epidermal growth factor receptor 2; Mutation + Amplification, concurrent mutation and amplification; 1L, first line; 2L, second line; TKI, tyrosine kinase inhibitor; PD-1, programmed cell death-1; PD-L1, programmed cell death-ligand1; CI, Confidence interval.

Of note, the efficacy of the RC48 combination treatment group showed better performance when compared with monotherapy (**Table 2**). The mPFS of patients who received RC48 combination therapy was 8.1 months (95% CI, 7.2-9.0 months; **Figure 2B**). The subgroup receiving RC48 in combination with platinum-based chemotherapy (with or without bevacizumab) achieved an impressive ORR of 71.4% (95%CI: 29.0-96.3%) and the mPFS was not reached. As shown in **Table 2**, the group of patients treated with RC48 plus *HER2* TKIs achieved a favorable outcome with an ORR of 50.0% (95%CI: 15.7-84.3%) and a mPFS of 7.2 months (95% CI, 3.6-10.8 months; **Supplementary Figure S1**). Patients receiving RC48 as a first-line treatment (n=8) showed the best efficacy, with an ORR of 62.5% (95% CI, 24.5-91.5%) and a mPFS of 8.1 months (95% CI,7.2-9.0 months). Patients undergoing second-line treatment (n=7) achieved an ORR of 57.1% (95% CI, 18.4-90.1%), and a mPFS of 7.2 months (95% CI, NA-NA). Patients in the >2L treatment group (n=7) had a mPFS of 7.4 months (95% CI, 3.4-11.4 months), although showing a lower ORR of 14.3% (0.4-57.9%), (**Table 2**, **Figures 2C**, **3**).

**Table 2:** Clinical response to RC48 in the overall *HER2* alterations NSCLC population and subgroups population.

	HER2 alteration status			Brain metastases		RC48 treatment line			Prior anti-PD-(L)1 therapy		Prior anti-*HER2* therapy		RC48 treatment regiment					Overall (N=22)
	Mut (N=15)	Amp (N=5)	Mut + Amp (N=2)	Yes (N=7)	No (N=15)	1L (N=8)	2L (N=7)	>2L (N=7)	Yes (N=7)	No (N=7)	Yes (N=7)	No (N=7)	RC48 alone	RC48 combined with				
													(N=1)	*HER2* TKIs (N=8)	Platinum +/-bevacizumab(N=7)	Antiangiogenic drugs (N=4)	PD-(L)1inhibitors +/- bevacizumab (N=2)	
ORR, n (%) [95% CI]	7(46.7) [21.3-73.4]	3(60) [14.7-94.7]	0 [0-84.2]	2(28.6) [3.7-71.0]	8(53.3) [26.6-78.7]	5(62.5) [24.5-91.5]	4(57.1) [18.4-90.1]	1(14.3) [0.4-57.9]	1(14.3) [0.4-57.9]	4(57.1) [18.4-90.1]	2(28.6) [3.7-71]	3(42.9) [9.9-81.6]	0 [0-97.5]	4(50.0) [15.7-84.3]	5(71.4) [29.0-96.3]	1(25) [0.6-80.6]	0 [0-84.2]	10(45.5%) [24.4-67.8]
CR	0	0	0	0	0	0	0	0	0	0	0	0	0	0	0	0	0	0
PR	7 (46.7)	3 (60.0)	0	2 (28.6)	8(53.3)	5(62.5)	4(57.1)	1 (14.3)	1(14.3)	9(60.0)	2(28.6)	8(53.5)	0	4(50.0)	5(71.4)	1(25.0)	0	10 (45.5)
SD	6(40.0)	2 (40.0)	2 (100.0)	3(42.8)	7(46.7)	3(37.5)	3(42.9)	4 (57.1)	5(71.4)	5(33.3)	3(42.9)	7(46.5)	0	4(50.0)	2(28.6)	2(50.0)	2(100.0)	10 (45.5)
PD	2(13.3)	0	0	2 (28.6)	0	0	0	2 (28.6)	1(14.3)	1	2(28.6)	0	1	0	0	1(25.0)	0	2 (9.1)
DCR, n (%) [95% CI]	13(86.7) [59.5-98.3]	5(100.0) [47.8-100]	2(100.0) [15.8-100]	5(71.4) [29.0-96.3]	15(100.0) [78.2-100]	8(100.0) [63.1-100]	7(100.0) [59.0-100]	5(71.4) [29.0-96.3]	6(85.7) [42.1-99.6]	6 (85.7) [42.1-99.6]	5(71.4) [29.0-96.3]	7(100.0) [59.0-100]	0 [0-97.5]	8(100.0) [63.1-100]	7(100.0) [59.0-100]	3(75.0) [19.4-99.4]	2(100.0) [15.8-100]	20(90.9) [70.8-98.9]

CR, complete response, PR, partial response, SD, stable disease, PD, progressive disease, ORR, objective response rate, DCR, disease control rate; ORR, objective response rate; ECOG, Eastern Cooperative Oncology Group; PS, performance status; *HER2*, human epidermal growth factor receptor 2; Mut, mutation; Amp, amplification; Mut+ Amp, concurrent mutation and amplification; 1L, first line; 2L, second line; TKI, tyrosine kinase inhibitor; PD-1, programmed cell death-1; PD-L1, programmed cell death-ligand1; CI, Confidence inter

Patients with prior anti-*HER2* therapy (n=7) responded to subsequent RC48-based anti-*HER2* treatment with an ORR of 28.6% (95% CI, 3.7-71%) and the mPFS of 7.2 months (95% CI, 3.2-11.2 months; **Figure 2D**). Among those previously treated with anti-PD-(L)1 inhibitors (n=7), the median treatment line was 3.5 (2-5 line), the ORR was 14.3% (95% CI, 0.4-57.9%), and the mPFS was 7.4 months (95% CI, 3.3-11.5 months; **Figures 2E**, **3**).

In patients present with baseline brain metastases, the ORR was 28.6% (95% CI, 3.7-71.0%), the DCR was 71.4% (95% CI, 29.0-96.3%; **Table 2**). The mPFS was 8.1 months (95% CI, 6.9-9.3 months) for patients presenting with baseline brain metastases, compared to 7.5 months (95% CI, 7.3-7.7 months) for those without baseline brain metastases. This comparison revealed no significant difference in mPFS between the two groups (*P*=0.503; **Figures 2F**, **3**).

Among NSCLC patients harboring *HER2* mutations (n=15), the RC48 treatment regimen had an ORR of 46.7% and a DCR of 86.7%. The *HER2*-amplified subgroup (n=5) showed an ORR of 60.0% and a DCR of 100.0%. In rare cases of concurrent amplification and mutation, the DCR reached 100.0% in both patients (**Table 2**, **Figure 3**). The mPFS of patients with *HER2* mutations, amplifications, and concurrent *HER2* mutation and amplification was 8.1 months (95% CI, 4.6-11.6 months), 9.4 months (95% CI, NA-NA) and 7.4 months (95% CI, NA-NA), respectively (**Figure 2G**). Median PFS was significantly prolonged in *HER2*-amplified patients, and no significant difference in mPFS was observed (*P*=0.73).

### 3.3 Safety

The duration of RC48 treatment ranged from 2 to 19 months with a median treatment period of 5.5 months. Importantly, none of the patients were found to have reduced or discontinued their medication due to side effects during treatment. Adverse events are detailed in **Table 3**. All patients reported at least one AE. The most common adverse events included hand-foot syndrome (54.5%), asthenia (50.0%), decreased white blood cell count (45.5%), and liver impairment (45.5%). Grade 3 adverse events occurred in one (4.5%) patient.

**Table 3:** Adverse events in the patients treated with RC48, n (%).

Event	All grades	Grade 1	Grade 2	Grade 3
*Decreased WBC count*	10 (45.5)	7 (31.8)	2 (9.1)	1 (4.5)
*Hand-foot syndrome*	12 (54.5)	12 (54.5)	0	0
*Asthenia*	11 (50.0)	11 (50.0)	0	0
*Liver impairment*	10 (45.5)	10 (45.5)	0	0
*Anemia*	8 (36.4)	7 (31.8)	1 (4.5)	0
*Diarrhea*	7 (31.8)	6 (27.3)	1 (4.5)	0
*Nausea or vomiting*	6 (27.3)	6 (27.3)	0	0
*Anorexia*	6 (27.3)	6 (27.3)	0	0
*Decreased platelet count*	3 (13.6)	2 (9.1)	1 (4.5)	0
*Rash*	2 (9.1)	2 (9.1)	0	0

AE, adverse event; WBC, white blood cell.

## 4 Discussion

*HER2*-targeted therapeutics have shown favorable antitumor efficacy, including tyrosine kinase inhibitors (afatinib, lapatinib, neratinib, and tucatinib), monoclonal antibodies (mAbs) (trastuzumab, pertuzumab, initumumab), and bispecific antibodies (23). mAbs precisely targets tumor surface antigens. However, its clinical efficacy is often inadequate because its lethality against cancer cells is not inadequate when using mAbs alone. ADC drugs are monoclonal antibodies loaded with a small toxin molecule which specifically targeting cancer cells and then produce a potent toxic effect. ADC drugs make up for the limitations of *HER2-*targeted therapies. Moreover, the ability to exert cytotoxic activity against antigen-negative cells of ADC drugs, also called the bystander effect, allows to overcome tumor heterogeneity (24). RC48 is a novel ADC drug comprised of disitamab coupling with the cytotoxic agent MMAE via a cleavable linker. It was well tolerated and showed promising efficacy in several *HER2*-positive cancers such as breast cancer (25), gastric cancer (26), and urothelial carcinoma (27).

To our knowledge, this is the first study conducted in a real-world setting to report the efficacy and safety of RC48 combination therapy in patients with advanced *HER2*-altered NSCLC. Our findings reveal that RC48 therapy yield a favorable clinical response with an ORR of 45.5%, a DCR of 90.9%, and a mPFS of 7.5 months among *HER2*-altered NSCLC. The combination therapy, particularly, showed enhanced effectiveness with an ORR of 47.6% and a mPFS of 8.1 months, underscoring the significant clinical benefits RC48 may offer to patients with *HER2*-altered NSCLC.

In our study, we observed that the combination therapy (RC48 with platinum-based chemotherapy, with or without bevacizumab) showed an encouraging ORR of 71.4%. Chemotherapy, as cytotoxic partners of ADCs, can not only interfere with the cell cycle but also modulate the expression of surface antigen targeted by ADCs. Platinum agents, which cause S phase cell cycle arrest and subsequent G2/M phase accumulation, seem to have a synergistic effect with microtubule inhibitors like MMAE within RC48 (28). This combination potential has also been illustrated by carboplatin with mirvetuximb soravtansine (Folate receptor (FR)a-DM4) (29). Furthermore, the well-balanced DAR design of 4 in RC48 also demonstrated a milder toxicity, making it an appealing and flexible companion of platinum in clinical settings (28, 30). As platinum-based chemotherapy still plays a fundamental role in NSCLC treatment, ADCs have the potential to enable the development of highly potent and safe combinations by replacing cytotoxic regimens based on a better understanding of mechanisms. Moreover, antiangiogenic agents may synergistically enhance the delivery of ADCs to tumor cells by normalizing the vasculature and improving treatment sensitivity (6, 31). The combination of anetumab ravtansine with bevacizumab has shown potent effects in ovarian cancer model (32). However, as far as we know, such a combination design has not been tested in clinical trials in NSCLC. Therefore, our data provides real-world evidence for future ADC clinical trial designs of similar combination schemes with antiangiogenesis in NSCLC.

The efficacy of RC48 in combination with *HER2*-TKIs also merits attention, with an ORR of 50.0% and a mPFS of 7.2 months observed in our study. The addition of *HER2*-ADC to pan-HER irreversible inhibitor *HER2*-TKIs, predominantly pyrotinib in our study, demonstrates synergistic efficacy in terms of ORR. In previous studies, pyrotinib monotherapy was shown to have an ORR of 30% as well as an mPFS of 6.9 months in *HER2*-altered NSCLC (12, 33). Co-administration of these agents may enhance the internalization of *HER2*-ADC, eliciting robust antitumor activity. Sub-therapeutic doses of TKI could be adequate for enhancing ADC-dependent cell death and tumor shrinkage, thereby reducing the adverse effects associated with the daily use of these agents (20). A concern with pyrotinib is its toxicity, which limits its clinical dosage. The most common TRAE observed with a dose of 400 mg of pyrotinib was diarrhea (92.6%), and the severity was positively in line with the dosage (20, 34). In our study, seven patients received pyrotinib, which was initiated at a low dose of 240 mg, and the dose was increased to 320 mg if no adverse reactions were observed. This combined regimen showed a manageable safety profile with discrete dose management based on patient tolerance. These data also suggest that the combination of RC48 with pyrotinib may be a promising therapeutic approach for *HER2*-altered NSCLC and warrants further comprehensive clinical evaluation.

Our study also differentiated the efficacy of RC48 among various *HER2* alterations and slight differences in efficacy were observed. For *HER2*-mutant NSCLC, the combination treatments exhibited an ORR of 46.7% and a median PFS of 8.1 months, comparable to current *HER2* ADCs like T-DXd and T-DM1 (19, 21). Those data suggest that RC48 presents a potential treatment option in patients with *HER2*-mutated NSCLC. In cases of *HER2* amplification, RC48 combination strategies showed promising results, with an ORR of 60.0% and a mPFS of 9.4 months. A preclinical study suggests that T-DXd could effectively inhibit the proliferation of *HER2*-amplified cells *in vitro* and *in vivo* (35). Other anti-*HER2* therapies include *HER2*-amplified NSCLC patients, such as T-DM1, which shows an ORR of 55% in 14 *HER2*-amplified patients enrolled in a phase II basket trial and pyrotinib, which also showed an ORR of 22.2% and a mPFS of 6.3 months in 22 patients (20, 34). These results suggest that *HER2* amplification may also be a target for anti-*HER2* therapy in NSCLC. However, there still requires large sample size research to prospectively identify optimal amplification cut-off value to target patients who can benefit most from anti-*HER2* therapies.

It is important to note that there was no statistically significant differences in our results, particularly in the mPFS comparisons between patients with and without baseline brain metastases and among different *HER2* alteration subgroups. It might be because the small sample size reduces the statistical power and may not represent a broader patient population

There are several limitations in this study. First, the retrospective nature of the study makes bias inevitable, and prospective studies are needed to validate these results. Second, although this study provided a comprehensive evaluation of all available treatment options and RC48 showed excellent antitumor activity in *HER2*-altered NSCLC, the small sample size reduces the statistical power and caution should be exercised in interpreting these results. Thirdly, this study was conducted during the COVID-19 pandemic, which may also have influenced outcomes, including delays in patient’s access to medical care, and delays in RC48 treatment. At last, the retrospective nature of the study may result in underreporting or recall bias in reporting AEs. Therefore, future prospective studies with more rigorous safety monitoring are needed to provide a more comprehensive understanding of the safety profile of RC48.

## 5 Conclusion

Despite the small sample size, this investigation introduces a viable therapeutic alternative for patients with advanced *HER2*-altered NSCLC, particularly through a regimen incorporating RC48 in conjunction with platinum-based chemotherapy, with or without bevacizumab. RC48-based therapies pave the way for new treatment in the case of HER2-amplified patients. Overall, the safety profile was well tolerated, and no dose reduction or discontinuation of treatment was found due to side effects. However, further studies with larger sample sizes are needed to confirm these preliminary findings. Future research on HER2-targeted ADCs should primarily focus on combination treatment strategies with other treatment modalities, to further improve patients’ outcomes.

## Data Availability

The original contributions presented in the study are included in the article/**Supplementary Material**. Further inquiries can be directed to the corresponding authors.
