# Supplementary material for: Efficacy of disitamab vedotin in non-small cell lung cancer with HER2 alterations: a multicenter, retrospective real-world study
Source: Front Oncol. 2024 Nov 6;14:1441025. doi: 10.3389/fonc.2024.1441025 (PMC11576286; doi:10.3389/fonc.2024.1441025)
Supplement: Supplementary file 1 [file Presentation1.pdf]

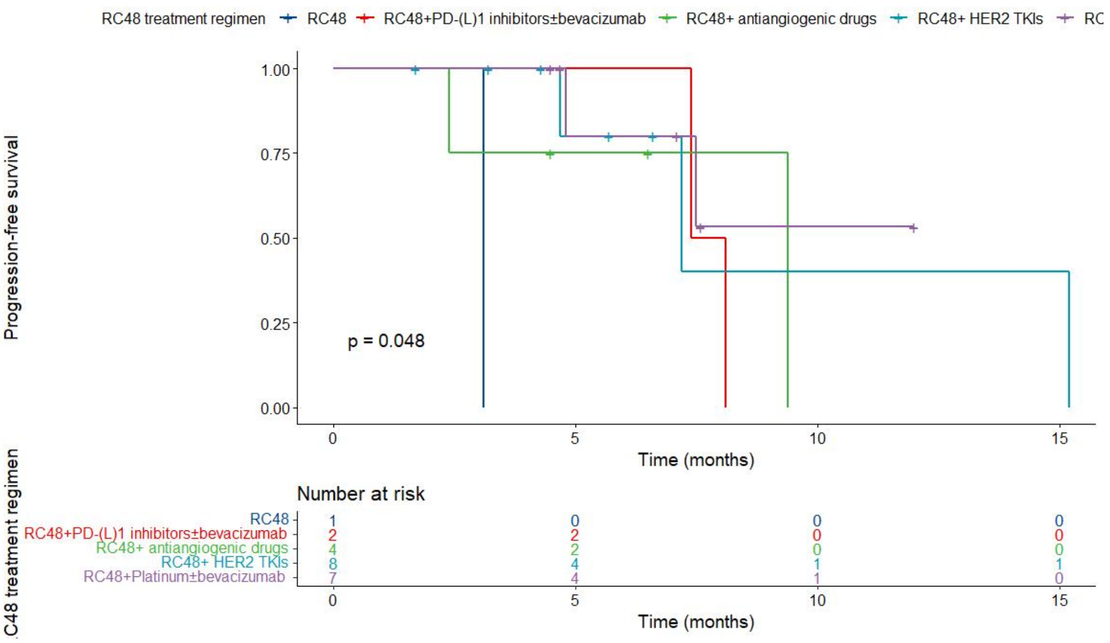

Supplemental Figure S1. Kaplan–Meier estimates of PFS in HER2-altered NSCLC patients with RC48 alone, RC48+PD-(L)1 inhibitors+/-bevacizumab, RC48+antiangiogenic drugs, RC48+HER2 TKIs, RC48+Platinum+/-bevacizumab; PD-1, programmed cell death-1; PD-L1, programmed cell death-ligand1; TKI, tyrosine kinase inhibitor.
